# Quality of websites about long-acting reversible contraception: a descriptive cross-sectional study

**DOI:** 10.1186/s12978-019-0835-1

**Published:** 2019-11-27

**Authors:** Catrin Eriksson, Matilda Skinstad, Susanne Georgsson, Tommy Carlsson

**Affiliations:** 1 Sophiahemmet University, Stockholm, Sweden; 2 The Swedish Red Cross University College, Huddinge, Sweden; 3 Department of Clinical science, Intervention and technology, Karolinska Institutet, Stockholm, Sweden; 4 Department of Women’s and Children’s Health, Uppsala university, MTC-huset, Dag Hammarskjölds väg 14B, 1 tr, SE-75237 Uppsala, Sweden

**Keywords:** Consumer health information, Long-acting reversible contraception, World wide web

## Abstract

**Background:**

Today, there are various short- and long-acting contraceptive alternatives available for those who wish to prevent unintended pregnancy. Long-acting reversible contraception are considered effective methods with a high user satisfaction. High-quality information about contraception is essential in order to empower individuals to reach informed decisions based on sufficient knowledge. Use of the Web for information about contraception is widespread, and there is a risk that those who use it for this purpose could come in contact with sources of low quality.

**Objective:**

The overarching aim was to investigate the quality of websites about long-acting reversible contraception.

**Methods:**

Swedish client-oriented websites were identified through searches in Google (*n* = 46 included websites). Reliability and information about long-acting reversible contraceptive choices were assessed by two assessors with the DISCERN instrument, transparency was analyzed with the Journal of the American Medical Association benchmarks, completeness was assessed with inductive content analysis and readability was analyzed with Readability Index.

**Results:**

The mean DISCERN was 44.1/80 (SD 7.7) for total score, 19.7/40 (SD 3.7) for reliability, 22.1/35 (SD 4.1) for information about long-acting reversible contraceptive choices, and 2.3/5 (SD 1.1) for overall quality. A majority of the included websites had low quality with regard to what sources were used to compile the information (*n* = 41/46, 89%), when the information was produced (*n* = 40/46, 87%), and if it provided additional sources of support and information (*n* = 30/46, 65%). Less than half of the websites adhered to any of the JAMA benchmarks. We identified 23 categories of comprehensiveness. The most frequent was *contraceptive mechanism* (*n* = 39/46, 85%) and the least frequent was *when contraception may be initiated following an abortion* (*n* = 3/46, 7%). The mean Readability Index was 42.5 (SD 6.3, Range 29–55) indicating moderate to difficult readability levels, corresponding to a grade level of 9.

**Conclusions:**

The quality of client-oriented websites about long-acting reversible contraception is poor. There is an undeniable need to support and guide laypersons that intend to use web-based sources about contraceptive alternatives, so that they may reach informed decisions based on sufficient knowledge.

## Plain English summary

Long-acting reversible contraception are safe and effective methods that prevent pregnancy. Devices placed in the uterus or under the skin of the arm release hormone or copper over several years, and do not require user compliance after insertion. When desired, the device can easily be removed by a health professional restoring the woman’s ability to get pregnant. Many who consider using contraception access the Web to read information, but little is still known about the information that they find. The aim of this study was to investigate the quality of websites about long-acting reversible contraception. We performed searches in Google to identify Swedish websites and found 46 that contained information directed to the general public. The websites were assessed with instruments widely used to appraise the quality of information developed for patients or clients. We also explored what topics that the websites discussed and evaluated their readability. The results showed that most websites were unreliable sources of information with low-quality information about contraceptive choices. The websites discussed a range of different topics, but many still lacked information about important aspects to consider when deciding which contraception to use such as associated costs, contraindications, and how it feels to have it in place. More than half of the websites were classified as difficult to read. Health professionals need to support and guide the public towards web-based information that will help them make well-grounded decisions about long-acting reversible contraception.

## Background

Long-acting reversible contraception (LARC) are methods that prevent unintended pregnancy through contraceptive activity that lasts for years and that can be removed when desired. These methods include the copper intrauterine device, the hormonal intrauterine system and the subdermal hormonal implant. Compared with short-acting contraception, LARC are more effective in preventing unintended pregnancies [1], are associated with greater client satisfaction, and result in higher continuation rates [2, 3]. Today, a large number of organizations and experts recommend LARC to those who desire effective contraception [1, 4–6]. Despite this, LARC is still underutilized, with reported rates between 15 and 24% worldwide [7–10]. The United Nations acknowledge regional differences in regard to prevalence of long-acting contraception, with higher prevalence in Asia and Northern America than in Africa and Europe [10]. Clients consider many factors when choosing between different contraceptive alternatives [11] and knowledge gained from information is a crucial aspect during decision-making [12]. Informed choices that relies in high-quality information are essential in the context of contraceptive counseling [13, 14], allowing clients to make choices based on relevant knowledge and consistent with their values. However, research indicate that laypersons have insufficient knowledge about LARC and underestimate its efficacy [7]. Studies show that those who seek contraceptive counseling want sufficient information about the available alternatives [8, 15] and indicate that many would consider LARC if provided with enough comprehensive information about the option [8].

Clients often seek information about LARC from a variety of sources during the decision-making process, including the Web [15, 16]. Indeed, population-based studies show that the general public frequently access the Web for health-related information, particularly women below 45 years of age [17]. The Web has the potential to be a large and accessible source for tailored information that could help clients reach informed decisions about contraceptive alternatives [18]. However, many distrust it as a source of information [19] and the substantial size implies it may be difficult to identify relevant high-quality information [20]. The unregulated structure of the Web makes it possible for anyone to publish content, possibly leading to dissemination of unsuitable low-quality information. Thus, health professionals in reproductive health services need to be prepared to discuss the use of web-based sources and offer recommendations on which websites are trustworthy and suitable to visit [21]. However, health professionals report that they lack sufficient time, resources and training to appropriately guide patients and clients towards high-quality web-based sources [18, 22].

An increasing amount of studies have articulated concerns concerning the quality of web-based information, including websites about reproductive health [23–25]. However, few studies have specifically investigated the quality of web-based information about LARC. According to one study investigating information about LARC for adolescents, few websites adhere to current clinical recommendations from the American College of Obstetricians and Gynecologists, the American Academy of Pediatrics, and the Society for Adolescent Health and Medicine, involving aspects such as indications, risks, and health benefits of LARC [26]. Another study published more than 15 years ago investigated information about the intrauterine device and observed that a significant proportion of the websites contained erroneous statements [27]. The widespread use of the Web for health-related information indicates a need for additional studies that investigate additional aspects related to the quality of websites about contraception. The overarching aim of this study was to investigate the quality of websites about long-acting reversible contraception. Specifically, we set out to assess the reliability, quality of information about long-acting reversible contraceptive choices, transparency, completeness and readability of client-oriented websites.

## Methods

### Study context

This study concerns Swedish websites. In Sweden, registered nurse-midwives and physicians prescribe and insert LARC. Swedish guidelines recommend that those who are of reproductive age and want to avoid a pregnancy should be offered information about the possibility to use LARC [5]. There is a very high Internet accessibility in Sweden and nearly all Swedes who are of reproductive age use the Web. Many use it to access and read information about health-related topics [28].

### Data collection

Swedish websites about LARC were identified through searches in Google, an online search engine. While there is a range of different search engines available on the Web, Google is the most used search engine, with 97% of the Swedish population between the ages of 16 and 65 years using it to search for web-based information [28, 29]. In line with recommendations in the literature [30], we set out to replicate search patterns among the general public. Thus, we designed the searches according to previous research about the search patterns of information consumers, namely using various types of search strings, screening the first ten links of the hit list before moving on to a new search, and limiting the data collection to the first web page presented when accessing the link presented in the web-based search engine [31–34]. Thus, we did not include any additional web pages found as links in the pages identified via the search engine. The research team designed 20 search strings that represent searches that laypersons use when searching for web-based information about LARC (Additional file 1). The two first authors (registered nurses and midwifery students) and the last author (specialist nurse, registered midwife and researcher) jointly decided which search strings to use. In line with previous studies about search procedures for health-related information [34], we designed the search strings to include general as well as specific terms for LARC (including brand names of the available and currently recommended LARC in Sweden [35]), and larger sentences as well as medical terminology. No quotation marks or other search operators were used.

**Fig. 1:**
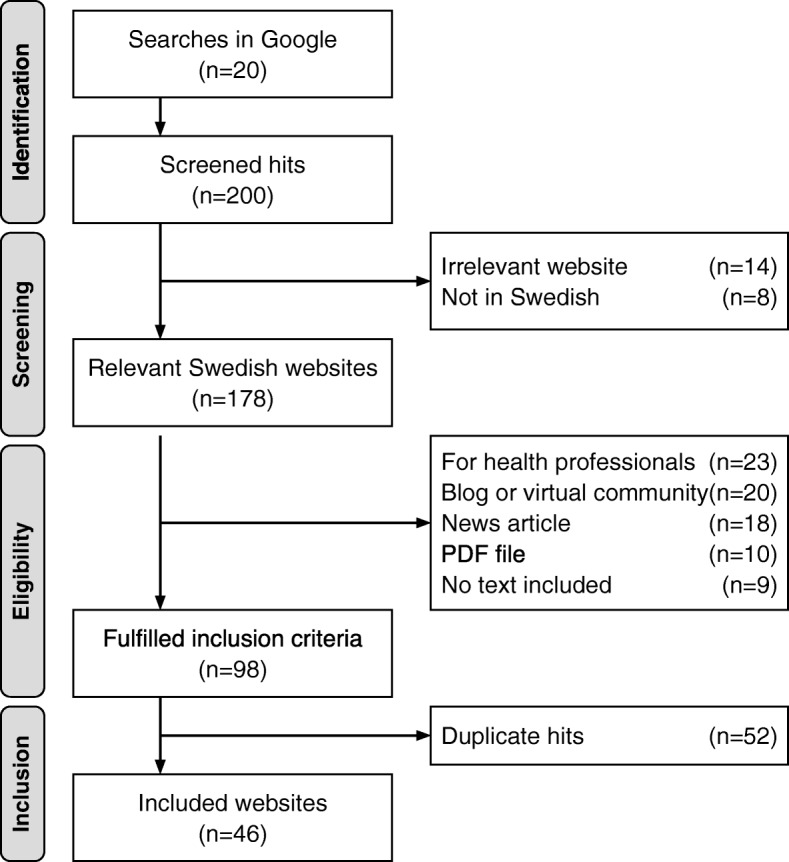
Search process for websites about long-acting reversible contraception

The searches were performed in January 2019 using the Web browser Safari and the first 10 hits of each hit list retrieved through the searches were screened for inclusion, resulting in 200 hits/links screened in total. To be included the websites needed to: (1) contain information about long-acting reversible contraception, (2) contain text-based information in Swedish, (3) be accessible without password requirements and (4) provide client-oriented information. Websites about LARC were excluded if they: (1) were written by laypersons in order to communicate and share personal experiences, including social media, discussion boards and blogs, (2) did not contain text-based material, (3) was a news article, (4) only led to a PDF-file that needed to be downloaded, and (5) provided information specifically for health professionals. Website domain was not given consideration when screening for inclusion. We identified 178 relevant Swedish websites that provided information about LARC. Of these, 98 were client-oriented websites that fulfilled the inclusion criteria. After correcting for duplicate hits (*n* = 52), 46 websites were included in the final sample. Details about the search process are presented in Fig. 1. In order to archive the content for later access, the included websites were saved with Webcite [36], an online archiving system for web-based references.

### Data analysis

The analysis was guided by current recommendations in the literature [30]. Website quality is a multidimensional concept [20, 37]. Thus, we assessed the websites with regard to five different quality aspects: reliability, information about long-acting reversible contraceptive choices, transparency, completeness, and readability. Descriptive statistics and interrater reliability was calculated with RStudio (version 1.0.143). The included websites were categorized depending on if they originated from (1) independent information websites or charities, (2) pharmaceutical companies, or (3) the government or health care system.

#### Reliability and information about long-acting reversible contraceptive choices

The DISCERN instrument was used to assess reliability and information about long-acting reversible contraceptive choices. The instrument is a valid and reliable tool [38, 39], developed by a range of specialists, consumer health information experts and lay representatives, and was funded by The British Library and the NHS Research & Development Programme. It has been used in many research studies investigating the quality of health-related information for patients and the general public [38]. The instrument includes subscales for reliability (eight questions), information about treatment choices (seven questions), and overall quality (one question). Each of the 16 questions is rated on a scale from 1 (no/low quality) to 5 (yes/high quality), resulting in a total score from 16 to 80. Subscale 1 (reliability) concern whether or not the publication can be trusted as a source of information about treatment choices, while subscale 2 (information about treatment choices) focus on specific details in the information about the choices described in the text and subscale 3 (overall quality) serves as an intuitive summary of the quality assessment according to the preceding questions in the instrument. In this study, information about treatment choices refers to information about long-acting reversible contraceptive choices (the copper intrauterine device, the hormonal intrauterine system, and the hormonal subdermal implant).

The first two authors, both female registered nurses and midwifery students with formal education in contraception and contraceptive counseling, assessed each included website independently. Interrater reliability was determined with intra-class correlation [40]. Reliability values below 0.4 indicate poor agreement, 0.4–0.59 indicate fair agreement, 0.6–0.74 indicate good agreement, and 0.75–1.0 indicate excellent agreement [41]. The mean scores of the two assessor’s scores were calculated for each included website and this score was used in the final analysis. The DISCERN instrument was developed so that website quality can be assessed similarly regardless of assessor background [39], and research has shown that professionals and laypersons rate quality of consumer health information similarly [42].

#### Transparency

Transparency was analyzed with the Journal of the American Medical Association (JAMA) benchmarks, presented by Silberg et al. [43]. The JAMA benchmarks assesses four basic criteria of website quality. The instrument includes questions concerning details about authorship (names, affiliations and credentials of authors), attribution (clearly stated references and sources for all content, and copyright information noted), disclosure (website ownership, sponsorship, advertising, underwriting, commercial funding, and support described, and potential conflicts of interest stated), and currency (date of production and updates stated). The last author analyzed the transparency.

#### Completeness

Completeness was assessed with inductive manifest content analysis [44], meaning that the identified categories were derived from the data. An inductive approach was used because we wanted the results to illustrate the complete set of data and not be constrained or colored by any preconceived theories or models. First, each website was read repeatedly to gain an understanding about the overall content. Second, meaning units were identified, defined as words, sentences or paragraphs that are related to each other through its content and context. Third, meaning units were arranged into categories, defined as collections of several meaning units that share a similar content. The number of websites represented in each category was then counted. The categorization was structured with the aid of NVivo (version 12). The first authors jointly conducted the content analysis and the last author scrutinized their analysis until consensus was achieved.

#### Readability

Readability was analyzed with the automated calculation Readability Index [Läsbarhetsindex] (LIX), commonly used for assessing the readability of Swedish texts. LIX scores range from > 25 (easiest texts) to > 60 (most difficult texts). Scores > 40 indicate that the text is too difficult for average persons to fully understand [45]. LIX scores < 10 are equivalent to first grade, while scores > 55 are equivalent to college. The corresponding grade levels of LIX scores are > 28 for elementary school (grades 1–5), 28–43 for junior high school (grades 6–9), 44–55 for senior high school (grades 10–12) and > 55 for college/university [46].

## Results

### Sample characteristics

The included sample originated from independent information websites or charities (*n* = 18, 39%), pharmaceutical companies (*n* = 15, 33%), and the Swedish government or health care system (*n* = 13, 28%). The top-level domains for the included websites were .se (*n* = 37, 80%), .com (*n* = 6, 13%) and .org (*n* = 3, 7%). The type of LARC covered by most websites was the hormonal intrauterine system (*n* = 28, 61%), followed by the subdermal implant (*n* = 25, 54%) and the copper intrauterine device (*n* = 22, 48%). A minority of the included websites covered all three types of LARC (*n* = 12, 26%) (Additional file 2).

### Reliability and information about long-acting reversible contraceptive choices

The interrater reliability was 0.56 for the DISCERN total score, 0.43 for reliability (subscale 1), 0.62 for information about long-acting reversible contraceptive choices (subscale 2) and 0.53 for overall quality (subscale 3), indicating fair to good overall agreement between the assessors. A closer inspection of interrater reliability revealed that the questions in the DISCERN instrument showed excellent (*n* = 5 of 16 questions), good (*n* = 3 of 16 questions), fair (*n* = 3 of 16 questions) and poor (*n* = 5 of 16 questions) agreement (Table 1). The mean score was 44.1 out of a total possible score of 80 (SD 7.7) for the total score, 19.7 out of a total possible score of 40 (SD 3.7) for reliability, 22.1 out of a total possible score of 35 (SD 4.1) for information about long-acting reversible contraceptive choices, and 2.3 out of a total possible score of 5 (SD 1.1) for overall quality (Table 1).

**Table 1:** Mean DISCERN scores of the included websites (*n* = 46) according to source of website and the overall interrater reliability (IRR)

Subscale	Question [score range]	Government or health care system (*n* = 13), M (SD)	Pharmaceutical company (*n* = 15), M (SD)	Independent information website or charity/ organization (*n* = 18), M (SD)	Overall (*n* = 46), M (SD)	IRR
Reliability	Clear aims [1-5]	2.8 (1.5)	3.2 (0.8)	2.6 (0.9)	2.8 (0.9)	0.11
	Achieve aims [1-5]	3.3 (1.4)	3.5 (0.9)	3.0 (0.9)	3.2 (1.1)	0.20
	Relevance [1-5]	3.9 (0.7)	3.8 (1.0)	3.1 (0.7)	3.6 (0.8)	0.67
	Clear what sources were used [1-5]	1.1 (0.3)	1.1 (0.3)	1.8 (1.1)	1.3 (0.8)	0.89
	Clear when the information was produced [1-5]	1.5 (0.5)	1.6 (0.5)	2.3 (0.7)	1.8 (0.7)	0.86
	Balanced & unbiased [1-5]	2.4 (0.5)	2.2 (0.6)	2.6 (0.6)	2.4 (0.6)	0.37
	Additional sources of support & information [1-5]	1.7 (0.6)	1.8 (0.8)	2.6 (1.5)	2.1 (1.1)	0.62
	Refer to areas of uncertainties [1-5]	2.3 (1.0)	2.7 (1.2)	2.6 (0.8)	2.5 (1.0)	0.59
	Total score [8-40]	18.9 (3.7)	19.1 (3.2)	20.5 (3.9)	19.7 (3.7)	0.43
Information about long-acting contraceptive choices	How long-acting contraception works [1-5]	3.9 (0.9)	3.2 (0.9)	3.2 (1.2)	3.4 (1.0)	0.75
	Benefits of long-acting contraception [1-5]	4.0 (1.1)	3.9 (1.2)	3.8 (1.4)	3.9 (1.2)	0.62
	Risks of long-acting contraception [1-5]	4.2 (1.4)	4.4 (1.2)	4.2 (1.0)	4.3 (1.2)	0.83
	What happen if no long-acting contraception is used [1-5]	3.0 (1.0)	2.7 (1.2)	2.6 (1.1)	2.8 (1.1)	0.14
	How long-acting contraception affect overall quality of life [1-5]	2.5 (0.7)	2.9 (0.8)	2.2 (0.5)	2.5 (0.7)	0.04
	That there is more than one long-acting contraceptive choice [1-5]	3.1 (1.4)	2.5 (1.2)	3.0 (1.1)	2.8 (1.2)	0.78
	Support for shared decision-making [1-5]	2.6 (0.9)	2.7 (0.9)	2.2 (0.8)	2.5 (0.9)	0.42
	Total score [7-35]	23.1 (3.6)	22.4 (4.5)	21.2 (4.1)	22.1 (4.1)	0.62
Overall quality	Total score [1-5]	2.3 (1.2)	2.3 (1.4)	2.3 (0.9)	2.3 (1.1)	0.53
Total	Total score [16–80]	44.3 (8.1)	44.5 (8.2)	44.0 (7.1)	44.1 (7.7)	0.56

**Fig. 2:**
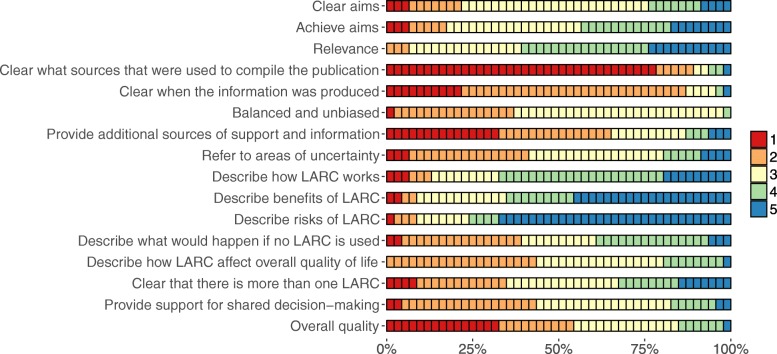
Distributions of DISCERN scores for the included websites (n = 46) about long-acting reversible contraception (LARC), ranging from 1 (no/low quality) to 5 (yes/high quality)

The majority of the included websites had low scores (i.e. a score of 1 or 2) for *clear what sources used to compile the information* (*n* = 41, 89%), *clear when information was produced* (*n* = 40, 87%), and *provide additional sources of support and information* (*n* = 30, 65%) (Fig. 2). The majority had high scores (i.e. a score of 4 or 5) for *describe risks of LARC* (*n* = 35, 76%), *describe how LARC works* (*n* = 31, 67%), and *describe benefits of LARC* (*n* = 30, 65%). The *overall quality* (subscale 3) was low for a majority of the websites (*n* = 25, 54%).

### Transparency

Table 2 presents the number of included web pages that adhered to the JAMA benchmarks. The highest frequency of websites that adhered to the criteria were found for the benchmark *authorship* (*n* = 18, 39%) and none of the included website adhered to the benchmark *disclosure*. With regard to number of websites that adhered to the benchmarks, 22 (48%) did not adhere to any benchmark, 23 (50%) adhered to one benchmark, and one website adhered to three benchmarks. When details about the author were provided, 17 (37%) websites stated that the information was written or reviewed by a health professional. Details about the date when the information was originally created was stated in 13 (28%) websites, in which the information was produced less than 1 year (*n* = 1, 2%), one year (*n* = 1, 2%), two years (*n* = 8, 17%), three years (*n* = 1, 2%), four years (*n* = 1, 2%) and 11 years (*n* = 1, 2%) before the data collection. Details about last update was stated in 20 (44%) websites, in which the information was updated less than 1 year (*n* = 3, 7%), one year (*n* = 13, 28%) and 2 years (*n* = 4, 9%) before the data collection.

**Table 2:** Proportion of included websites (*n* = 46) that adhered to the JAMA benchmarks

Benchmark	Aspects of benchmark disclosed in the web page	Government or health care system, n (%)	Pharmaceutical company, n (%)	Independent information website or charity/organization, n (%)	Total sample, n (%)
Authorship	Name of author	9 (69)	0 (0)	9 (50)	18 (39)
	Author credentials	9 (69)	0 (0)	9 (50)	18 (39)
	Author affiliation	9 (69)	0 (0)	9 (50)	18 (39)
	Adhered to all aspects of benchmark	9 (69)	0 (0)	9 (50)	18 (39)
Attribution	References	0 (0)	1 (7)	7 (39)	8 (17)
	Copyright information	4 (31)	11 (73)	14 (78)	29 (63)
	Adhered to all aspects of benchmark	0 (0)	0 (0)	7 (39)	7 (15)
Disclosure	Site ownership	13 (100)	15 (100)	15 (83)	43 (94)
	Sponsorship, advertising, underwriting, commercial funding arrangements or support	11 (85)	4 (27)	10 (56)	25 (54)
	Conflicts of interest	0 (0)	0 (0)	0 (0)	0 (0)
	Adhered to all aspects of benchmark	0 (0)	0 (0)	0 (0)	0 (0)
Currency	Date created	0 (0)	4 (27)	9 (50)	13 (28)
	Date updated	7 (54)	3 (20)	10 (56)	20 (44)
	Adhered to all aspects of benchmark	0 (0)	0 (0)	1 (6)	1 (2)

### Completeness

In total, 23 categories that portray completeness were identified (Table 3). The most common categories were information about *contraceptive mechanism* (*n* = 39, 85%), *insertion of LARC* (*n* = 37, 80%), *potential adverse reactions, risks and complications* (*n* = 36, 78%), *benefits associated with LARC* (*n* = 35, 76%), and *duration of LARC* (*n* = 34, 74%). The least common categories were information about *interactions with other drugs* (*n* = 7, 15%), *how it physically feels to have intrauterine LARC in place* (*n* = 7, 15%), *expected effects during the initial period with LARC* (*n* = 5, 11%), *how to check the position of LARC* (*n* = 5, 11%), and *when contraception may be initiated following an abortion* (*n* = 3, 7%). Additional file 3 presents the content of the categories illustrating completeness.

**Table 3:** Identified categories portraying the topics covered in the Swedish websites about long-acting reversible contraception (*n* = 46)

Category	n (%)
Contraceptive mechanism	39 (85)
Insertion of LARC	37 (80)
Potential adverse reactions, risks and complications	36 (78)
Benefits associated with LARC	35 (76)
Duration of LARC	34 (74)
Configuration and appearance	34 (74)
Contraceptive efficacy	32 (70)
Possible disadvantages of LARC	30 (65)
Expected effects that hormonal LARC has on menstruation	26 (57)
Return of fertility after removal	23 (50)
Removal of LARC	18 (39)
Referral and support for shared decision-making	17 (37)
Use following childbirth and during breast feeding	16 (35)
Associated costs	13 (28)
Actions if pregnancy occurs	11 (24)
Accessibility and which health professionals prescribe LARC	11 (24)
Contraindications	10 (22)
Interactions with other drugs	7 (15)
How it physically feels to have intrauterine LARC in place	7 (15)
Expected effects during the initial period with LARC	5 (11)
How to check position of LARC	5 (11)
When contraception may be initiated following an abortion	3 (7)

### Readability

The mean LIX score was 42.5 (SD 6.3, Range 29–55), and the clear majority of websites had LIX scores ranging between 30 and 49, indicating moderate to difficult readability levels (Table 4). Most websites originating from government or health care system had moderate readability levels (*n* = 11 of 13 websites, 84%), while websites originating from pharmaceutical companies and independent information websites or charities/organizations had difficult readability levels (*n* = 10 of 15 websites, 66% and *n* = 11 of 18 websites, 61%).

**Table 4:** Readability scores of the included websites (*n* = 46)

LIX score	What score represent	Equivalent grade level^a^	Government or health care system, n (%)	Pharmaceutical company, n (%)	Independent information website or charity/organization, n (%)	Total sample, n (%)
< 25	Easy-to-read, children’s books	1–4	0 (0)	0 (0)	0 (0)	0 (0)
25–29	Easy level, fiction	5–6	1 (8)	0 (0)	0 (0)	1 (2)
30–39	Moderate level, newspapers	6–8	10 (76)	4 (27)	3 (17)	17 (37)
40–49	Difficult level, official texts	9–11	1 (8)	10 (66)	11 (61)	22 (48)
50–60	Very difficult level, bureaucratic texts	11-College	1 (8)	1 (7)	4 (22)	6 (13)
> 60	Highest difficulty level, dissertations	College	0 (0)	0 (0)	0 (0)	0 (0)

^a^ [46]

## Discussion

The overarching aim of this study was to investigate the quality of websites about LARC. Based on our assessments, information about long-acting contraceptive choices and the reliability was low, most websites did not include sufficient details about transparency, there was a wide range regarding completeness, and the majority had moderate or difficult readability levels.

Few studies have systematically assessed the quality of web-based information about LARC, even though many clients access such information via the Web to learn more about contraceptive alternatives [15]. The quality deficits observed in this study further illustrate the problematic situation reported regarding web-based information about LARC [26, 27]. Previous studies have focused on other sets of quality criteria to assess information about LARC for adolescents [26] and the intrauterine device [27]. Studies have previously reported quality-related issues on websites about contraception with regard to accuracy [25, 27, 47], credibility [47], currency [27], presence of misleading information [25, 27], content [26], and readability levels corresponding to above elementary school [48]. A recent similar study investigating quality with the DISCERN instrument and JAMA benchmarks show comparable quality problems as our study for websites about combined oral contraceptives [48], further strengthening our findings and indicating that the investigated quality criteria are poor in a wider perspective within the context of contraception. This study adds new knowledge about web-based information, relevant for health professionals who work with contraceptive counseling.

We observed low quality for all the investigated quality aspects, which illustrates the need for improvement of widespread public information about long-acting contraception [7, 8]. Our study indicates that clients who rely on web-based sources will have impaired capability to reach informed choices about LARC. This is problematic considering the prevalent use of the Web for information about reproductive health [16, 49] and the importance of informed choices in regard to contraception [14]. The results of this study further illustrate the risk of misinformation on the Web with a high probability that clients are introduced to information of low quality when accessing the Web [50]. Details about when the information was produced were presented in a minority of the included websites, and a proportion of the websites was produced several years before data collection. Guidelines about LARC have changed drastically in the last decade, from only being offered to selective populations to becoming a first-line option, including being an option for nullipara of all ages [51]. Interestingly, the low quality scores were found across all types of websites, and government-associated websites had lowest reliability. The findings call attention to the risk that clients who seek web-based information about LARC are presented with outdated, insufficient and unbalanced information, regardless of which website affiliation they turn towards. Thus, information consumers who rely on web-based sources may not be empowered in their decision-making whether or not these contraceptive methods are of interest to them. Health professionals need to be mindful of this risk and bring this up for discussion during contraceptive counseling.

The findings concerning low quality adds to the existing literature that illustrate poor quality of health-related information on the Web [23–25, 52, 53]. This issue has continuously been raised by researchers and is of great concern today. Despite the growing body of evidence concerning quality deficits [23–25, 52, 53], studies report that patients regard the quality of websites as adequate or high [54] and experience the Web as a convenient and comprehensive source for information [55]. During counseling with health professionals, many patients and clients do not discuss the information they find on the Web. Other patients and clients decide to solely rely on information found via the Web without meeting a health professional at all [54]. In addition, health professionals describe that informing patients and clients about web-based information is a demanding and time-consuming challenge [22, 56], to such an extent that they may dismiss the need to discuss this subject during consultations [56–58]. There is an acknowledged need for initiatives that promote communication about online sources and a need for development of guidelines about web-based information [22]. Our findings illustrate the considerable efforts needed among the public in order to successfully identify high-quality websites about LARC. There is a need for future studies that investigate how to appropriately guide the public to the most suitable sources.

There are methodological limitations of this study that need to be addressed. We used Google to identify websites that clients find when searching for information about LARC, which is the most used search engine [29]. We chose Google as search engine to achieve a sample that portrays websites accessed by most Internet users. Our searches resulted in many duplicate hits, indicating saturation with regard to the achieved sample. We screened the first 10 hits of each search strings, based on the fact that laypersons very seldom access links beyond this number [31, 32, 34]. Search engines such as Google utilize complex algorithms and indexing systems today, resulting in effective searches that rank search results based on a great number of aspects [59]. To verify the initial searches we performed additional searches in October 2019 using another web browser (Google Chrome), set as incognito/private and applying the same search strings as the initial searches. The renewed searches only revealed two new websites that fulfilled the inclusion criteria, which upon inspection are highly similar to the websites originally identified in the initial searches. This further strengthens that the initial searches adequately represent what websites members of the public find when searching for web-based information about LARC. We excluded links in the hit list that directly led to a Portable Document Format (PDF), because we did not define this as a website but rather written information more closely resembling a brochure or a book. Readers need to take into consideration that the results of this study concern websites with text-based information, and not any other types of documents or media found online. We cannot make any certain claims about what websites that laypersons come in contact with when they use different search strategies, and the findings should be interpreted based on the search engine and search strings used in this study. Moreover, the included websites were all written in Swedish. It is possible that websites in other languages could have other quality scores. The generalizability needs to be considered with this in mind. While it is probable that the Swedish population read websites in other languages, it is also possible that consumers living outside of Sweden who can read Swedish would access the included websites. The Web is constantly growing and expanding. This was a cross-sectional study of Swedish websites, and thus, reflects the current quality of web-based sources. We encourage future additional studies that investigate quality of websites about LARC in other countries and languages.

Quality was assessed using three instruments and one inductive analysis. Combined, these methods measure five different aspects of the multidimensional concept information quality [37]. Completeness was inductively explored with manifest content analysis, a method that is suitable to use without a preconceived theory or model to systematically explore patterns in text-based data [44, 60]. While all these aspects strengthen the results, there are still other aspects of website quality that was not explored in this study, e.g. accuracy and usability [37]. We analyzed the websites with a total of three assessors that rated different aspects of quality. The assessments with DISCERN, conducted by two assessors who separately rated the websites, showed fair to good interrater agreement for the subscales. A closer inspection of the interrater reliability of each question in the instrument revealed fair to excellent interrater reliability for 11 of the 16 questions and poor reliability for 5 questions. The questions with poor interrater reliability concerned presentation of aims, balance and bias, what would happen if no LARC is used, and how long-acting contraceptive choices affect overall quality of life. These quality aspects need to be interpreted with more caution because of the poor agreement between the assessors. We cannot dismiss the possibility that clients would rate the websites differently than the assessors, all with backgrounds as health professionals. More studies that encompass other aspects of websites quality and use other types of assessors are needed, to gain a comprehensive and nuanced understanding about quality of websites about LARC.

## Conclusion

The quality of Swedish client-oriented websites about long-acting reversible contraception is insufficient with regard to reliability, quality of information about long-acting reversible contraceptive choices, transparency, completeness of information and readability. While the quality of information about risks and benefits seems adequate, most websites need to have their quality improved. Developers of web-based information about long-acting reversible contraception need to make sure that the websites are readable and transparent in regard to authorship, attribution, disclosure and currency. Website developers should also include details on the reliability of the information and strive to publish nuanced comprehensive high-quality information about the different alternatives available for those who consider long-acting reversible contraception. There is an undeniable need to support and guide clients that intend to use web-based sources about contraceptive alternatives, so that they may reach informed choices based on sufficient knowledge. Future studies should focus on methods that aim to aid health professionals communicate recommendations for high-quality web-based sources. There is an overarching need for development of interventions that raise the quality of websites about long-acting reversible contraception.

## Supplementary information

**Additional file 1.** Search strings and included hits.

**Additional file 2. **Long-acting reversible contraception covered in the included websites (*n* = 46).

**Additional file 3. **Content of categories illustrating completeness in included websites (*n* = 46).

## Data Availability

The datasets used and/or analysed during the current study are available from the corresponding author on reasonable request.

## Abbreviations

JAMA: Journal of the American Medical Association
LARC: Long-acting reversible contraception
LIX: Readability Index [Swedish: Läsbarhetsindex]
