# Supplementary material for: Quality of websites about long-acting reversible contraception: a descriptive cross-sectional study
Source: Reprod Health. 2019 Nov 27;16:172. doi: 10.1186/s12978-019-0835-1 (PMC6882246; doi:10.1186/s12978-019-0835-1)
Supplement: Supplementary file 1 — Additional file 1. Search strings and included hits. [file 12978_2019_835_MOESM1_ESM.pdf]

Multimedia Appendix 1. Search strings and included hits.

Search string in Swedish	Search string translated in English	Total hits	Included hits	
			Unique	Duplicate
Preventivmedel stav	*Contraception rod*	19,800	8	0
P-stav[a]	[P-stav][a]	36,700,000	4	4
Preventivmedel implantat	*Contraception implant*	22,200	4	2
Nexplanon[b]	*Nexplanon*[b]	702,000	0	2
Hormonspiral	*Hormonal IUD*	619,000	8	0
Kopparspiral	*Copper IUD*	798,000	4	0
Preventivmedel spiral	*Contraception IUD*	118,000	1	6
Jaydess[b]	*Jaydess*[b]	2,140,000	1	0
Mirena[b]	*Mirena*[b]	9,780,000	3	0
Kyleena[b]	*Kyleena*[b]	243,000	1	0
Långverkande reversibelt preventivmedel	*Long-acting reversible contraception*	4,560	2	0
Preventivmedel	*Contraception*	2,930,000	3	2
Hur fungerar långverkande reversibla preventivmedel	*How does long-acting reversible contraception work*	2,750	0	2
Hur fungerar hormonspiral	*How does the hormonal IUD work*	138,000	0	7
"Hur fungerar kopparspiral	*How does the copper IUD work*	202,000	2	5
Hur fungerar p-stav[a]	*How does the* [p-stav] *work*[a]	118,000	1	7
Vilka långverkande preventivmedel finns	*What long-acting contraception are available*	24,000	3	4
Vad är långverkande preventivmedel	*What is long-acting contraception*	15,600	0	4
Hur sätter man in en spiral	*How do you insert a IUD*	281,000	1	4
Hur sätter man in en p-stav[a]	*How do you insert a* [p-stav][a]	159,000	0	3
**Total for all search strings**			**46**	**52**

[a] P-stav: Common Swedish term for the subdermal implant
[b] Brand names of available and recommended LARC
