# Supplementary material for: Quality of websites about long-acting reversible contraception: a descriptive cross-sectional study
Source: Reprod Health. 2019 Nov 27;16:172. doi: 10.1186/s12978-019-0835-1 (PMC6882246; doi:10.1186/s12978-019-0835-1)
Supplement: Supplementary file 2 — Additional file 2. Long-acting reversible contraception covered in the included websites (n = 46). [file 12978_2019_835_MOESM2_ESM.pdf]

Multimedia Appendix 2. Long-acting reversible contraception (LARC) covered in the included websites (n=46).

Contraception covered	Government or health care system, n (%)	Pharmaceutical company, n (%)	Independent information website or charity/organization, n (%)	Total sample, n (%)
Hormonal intrauterine system	8 (62)	9 (60)	11 (61)	28 (61)
Copper intrauterine device	7 (54)	6 (40)	9 (50)	22 (48)
Subdermal implant	8 (62)	6 (40)	11 (61)	25 (54)
All types of LARC	5 (38)	2 (13)	5 (28)	12 (26)
