# Supplementary material for: Quality of websites about long-acting reversible contraception: a descriptive cross-sectional study
Source: Reprod Health. 2019 Nov 27;16:172. doi: 10.1186/s12978-019-0835-1 (PMC6882246; doi:10.1186/s12978-019-0835-1)
Supplement: Supplementary file 3 — Additional file 3. Content of categories illustrating completeness in included websites (n = 46). [file 12978_2019_835_MOESM3_ESM.pdf]

Multimedia Appendix 3. Content of categories illustrating completeness in included websites (n=46).

**Category**	**Content**	**n (%)**
Contraceptive mechanism	How contraception works, e.g., local or systemic effect, effect on ovulation	39 (85)
Insertion of LARC	Preparations before insertion, pain relief during insertion, where LARC is placed, what type of health professionals that perform the insertion	37 (80)
Potential adverse reactions, risks and complications	Acne, allergic reactions, alopecia, anxiety, arthralgia, breast growth and tenderness, diarrhea, dysmenorrhea, ectopic pregnancy, edema, expulsion, fatigue, flatulence, headaches, heightened appetite, hirsutism, hot flashes, hypertension, infections, irritability, loss of libido, menorrhagia, nausea and vomiting, obstipation, ovarian cysts, pain, paresthesia, pruritus, psychological reactions, scars, seizures, sleeplessness, unintended pregnancy, uterine perforation, vaginal discharge, vasovagal syncope, vulvovaginitis, weight gain	36 (78)
Benefits associated with LARC	Benefits of the LARC, e.g., long-term contraception, no need to remember once it is in place, low hormonal dose, it is not visible, that it can be used by nullipara	35 (76)
Duration of LARC	How many years that LARC has contraceptive effect before it needs to be replaced	34 (74)
Configuration and appearance	How it looks, what material is it made of, how does it stay in place	34 (74)
Contraceptive efficacy	How well LARC protects against unintended pregnancy	32 (70)
Possible disadvantages of LARC	No STI prevention, discomfort during insertion, need help from midwife or physician, foreign device placed in the body, amenorrhea for those who desire menstruation	30 (65)
Expected effects that hormonal LARC has on menstruation	How menstruation is changed when hormonal LARC is in place (metrorrhagia/spotting or amenorrhea)	26 (57)
Return of fertility after removal	How long before fertility return once LARC has been removed	23 (50)
Removal of LARC	How to make an appointment for removal, when removal is due after insertion, how the removal is performed, if pain relief can be used during removal	18 (39)
Referral and support for shared decision-making	Encouragement to talk with health professionals about LARC, the possibility and rights to attend contraceptive counseling, where to turn for contraceptive counseling	17 (37)
Use following childbirth and during breast feeding	When LARC can be inserted following childbirth, possible effects on breast milk, effects LARC have on babies who nurse	16 (35)
Associated costs	Associated costs for different age groups	13 (28)
Actions if pregnancy occurs	The need to remove LARC if pregnancy occurs during contraceptive treatment	11 (24)
Accessibility and which health professionals prescribe LARC	The need for prescription, the fact that only midwives and physicians can prescribe the contraception, collecting LARC at the pharmacy	11 (24)
Contraindications	Diseases that prevent use, current pregnancy is a contraindication	10 (22)
Interactions with other drugs	How hormonal LARC effects other drugs and how drugs effect LARC	7 (15)
How it physically feels to have intrauterine LARC in place	If and how intrauterine LARC should be felt in everyday life, if and how the cords from the intrauterine device can be felt by themselves or by a partner during sex	7 (15)
Expected effects during the initial period with LARC	The need for a time period before it is possible to know the extent of possible adverse reactions	5 (11)
How to check position of LARC	How and when to check the placement of intrauterine LARC by feeling the cords	5 (11)
When contraception may be initiated following an abortion	If and when it is possible to have LARC inserted following an abortion	3 (7)

LARC = Long-acting reversible contraception
STI = Sexually transmitted infections
